# Spatial memory is as important as weapon and body size for territorial ownership in a lekking hummingbird

**DOI:** 10.1038/s41598-018-20441-x

**Published:** 2018-01-31

**Authors:** Marcelo Araya-Salas, Paulina Gonzalez-Gomez, Katarzyna Wojczulanis-Jakubas, Virgilio López, Timothy F. Wright

**Affiliations:** 1 Laboratory of Ornithology, Cornell University, Ithaca, NY USA; 2 Escuela de Biología, Universidad de Costa Rica, San Pedro, San José, Costa Rica; 3 Department of Neurobiology, Physiology and Behavior, University of California Davis, Davis, CA USA; 4 Department of Vertebrate Ecology and Zoology, University of Gdansk, Gdansk, Poland; 5 Department of Kinesiology, University of Connecticut, Connecticut, CT USA; 6 Department of Biology, New Mexico State University, Las Cruces, NM USA; 7 Universidad Autonoma de Chile, Av. Pedro de Valdivia 425, Providencia, Santiago Chile

## Abstract

Advanced cognitive abilities have long been hypothesized to be important in mating. Yet, most work on sexual selection has focused on morphological traits and its relevance for cognitive evolution is poorly understood. We studied the spatial memory of lekking long-billed hermits (*Phaethornis longirostris*) and evaluated its role in lek territory ownership, the magnitude of its effect compared to phenotypic traits expected to influence sexual selection, and whether its variation is indicated in the structure of mating vocal signal. Spatial memory (the ability to recall the position of a rewarding feeder) was compared between “territorial” and “floater” males. Interestingly, although spatial memory and body size both positively affected the probability of lek territory ownership, our results suggest a stronger effect of spatial memory. Bill tip length (used as weapon in agonistic interactions) also showed a positive but smaller effect. Load lifting during vertical flight, a measure of physical performance relevant to agonistic interactions, had no effect on territory ownership. Finally, both body size and spatial memory were indicated in the structure of male song: body size negatively correlated with song lowest frequency, while spatial memory positively predicted song consistency. Together, our findings lend support for cognition as a sexual selection target.

## Introduction

Elucidating the forces that have favored the evolution of advanced cognitive abilities remains central to understand some of the most elaborate behaviors found in nature, including ones that characterize our own species^1^. Cognition, broadly defined as the ability to acquire, process, retain and use information^2^, allows animals to finely tune behavior during their life time in response to rapidly changing environments, which provides an obvious fitness advantage. Its adaptive value is further supported by the typically large investment of energy and tissues in organs involved in complex cognitive tasks^2,3^. Nonetheless, until recently, cognitive research has focused mainly on broad evolutionary patterns, underlying mechanisms or ecological drivers of cognition and little is still known on the role of intra-specific interactions on the evolution of this fascinating trait^4^.

Sexual selection, a strong evolutionary force responsible for some of the most elaborate traits in nature^5^, has long been an obvious candidate for a selective pressure favoring advanced cognition^6^. A preference for cognitively superior mates could result in direct benefits as they may provide better resources for rearing offspring. If cognitive skills are heritable, as has been shown in some species^7,8^, indirect benefits could also be gained in the form of “good genes” for better cognitive abilities that can be passed to the offspring^9^. In addition, the complexity of many courtship displays, which often involve elaborated motor patterns (e.g. long song repertoires^10^, coordinated visual displays^11^) or challenging behavioral tasks (e.g. bower building in bowerbirds^12^), suggest they are cognitively demanding behaviors and could serve as honest indicators of cognitive abilities for mate choice or intra-sexual competition. Surprisingly, little is known on the role of sexual selection on the evolution of complex cognition.

Sexual selection favors traits that increase the reproductive output either by direct selection from the opposite sex (i.e. female choice) or by improving the access to mates through intra-sexual competition^5^. Cognition could provide an advantage for either of these forms of sexual selection, although most work has focused on female choice^9,13^. Nonetheless, evidence indicating that cognitive traits are actually favored by sexual selection is still scarce^4,9^. Female preference for individuals that performed better in cognitive tests has been shown in guppies (*Poecilia reticulata*, learning ability^14^), satin bowerbirds (*Ptilonorhynchus violaceus*^15,16^; but see^4,13,17^ for an alternative interpretation), meadow voles (*Microtus pennsylvanicus*^18^) and humans^19^. Evidence linking cognition and intra-sexual competition is rarer still. Performance in a problem solving task and the ability to monopolize food resources was inversely correlated in great tits (*Parus major*^20^, although the cognitive dimension of this behavior has been debated^13^). Primate species with stronger male-male competition have more developed brain structures involved in sensory motor skills and aggression^21^, which indirectly suggests that intra-sexual selection shapes cognitive traits.

The link between cognition and mating signals has been studied in more depth^9^. Research has focused on learned vocalizations because their cognition-mediated development potentially provides a mechanism to reliably indicate overall cognition^22–24^, but support for this link has been mixed. Male zebra finches (*Taeniopygia guttata*) that were more proficient at a novel foraging task had also more song elements but did not differ in the number of unique elements^25^. However, no link between cognitive performance and song complexity was found when sociable zebra finches were evaluated in flocks rather than individually^26^. Song bout length was positively correlated to spatial learning but negatively to social learning in European starlings (*Sturnus vulgaris*^27^). In song sparrows (*Melospiza melodia*) repertoire size was correlated to performance in a detour-reaching task^28^ but inversely correlated to success in color reversal^28^ and spatial memory tasks^29^. However, a third study on song sparrows that evaluated the link between performance in the same three cognitive tasks and song learning accuracy found positive correlations to color reversal and spatial learning performance but negative correlations to success in detour-reaching and novel-foraging tasks^30^.

Hummingbirds offer a particularly compelling group to examine the link between cognition and mating behavior^31^. They are among the smallest of vertebrates while employing some of the most energetically costly forms of locomotion^32,33^, and thus face extreme challenges in maintaining energy balance^34,35^. They maintain their high-energy lifestyle largely with constant access to nectar^36^, a resource that varies in quality over space and time^37^. In response they have evolved advanced cognitive abilities including fine-tuned discrimination^38–42^, detailed spatial memory, and episodic-like memory, all of which allows them to maximize foraging efficiency^31,37,43^. Traplining foraging (i.e. the use of flowers dispersed across the habitat in a route like fashion), exhibited by many hermit hummingbird species (subfamily Phaethornitinae), involves learning the location of inflorescences and rewarding flowers within those inflorescences across extended forest areas^44^. Given the high cost of flying for hummingbirds, mistakenly visiting unrewarding flowers would imply a higher energetic cost for trapliners as they visit patchy distributed flowers^31^, in turn suggesting selection for cognitive skills that reduce mistakes^45^. In addition, most hermits exhibit a lek mating system with strong male competition for mating territories^46^. Cognitive abilities that improve foraging efficiency could provide a key advantage for obtaining and defending lek space. Furthermore, males are well known for their active vocal signaling^47^, which involves advanced vocal learning skills^48,49^, providing a potential link between cognition and mating signals.

In this study we assessed the relation between cognitive abilities and the performance at reproductive grounds in male long-billed hermits (*Phaethornis longirostris*). Males form leks of 5–15 territories where they display for 6–8 hours a day for during the 8 month breeding season^50^, an extraordinary effort only sustained by males in good condition, resulting in an annual turnover of 48% at leks^50^. Songs are the primary mating signal, which they sing at a high rate throughout the day and can be learned throughout lifetime (ref.^48^; Video S1). In addition, the rarity and unpredictability of female visits (~1 visit per lek per day^50^) suggests males are under strong selection to be present, displaying and prepared to mate throughout the long mating season.

We evaluated a cognitive trait with a clear fitness value in this species, spatial learning of nectar resources. We conducted a spatial learning task on free-living hummingbirds^40,41^, and related its variation to both the ability to acquire and defend a lek territory (as a proxy of reproductive success) and to the structure of mating vocal signals (as a possible honest indicator of cognitive abilities). The latter association could provide an honest index signal for male-male competition and female choice^22–24^. In addition, the effect of spatial memory was contrasted to those of physical condition-related phenotypic traits such as body size, size of the bill tip (a weapon in male-male agonistic interactions), and load lifting during vertical flight (a measure of physical performance), as these represent common correlates of dominance and reproductive success^5,51–53^, particularly in lekking species^54,55^. Our findings indicate that spatial memory could play a key role in territory acquisition and defense, and suggest that its effect is more pronounced than those of other phenotypic traits. They also suggest male song reliably indicates spatial learning and memory in this species, further highlighting the relevance of cognitive traits in mating behavior.

## Results

**Figure 1:**
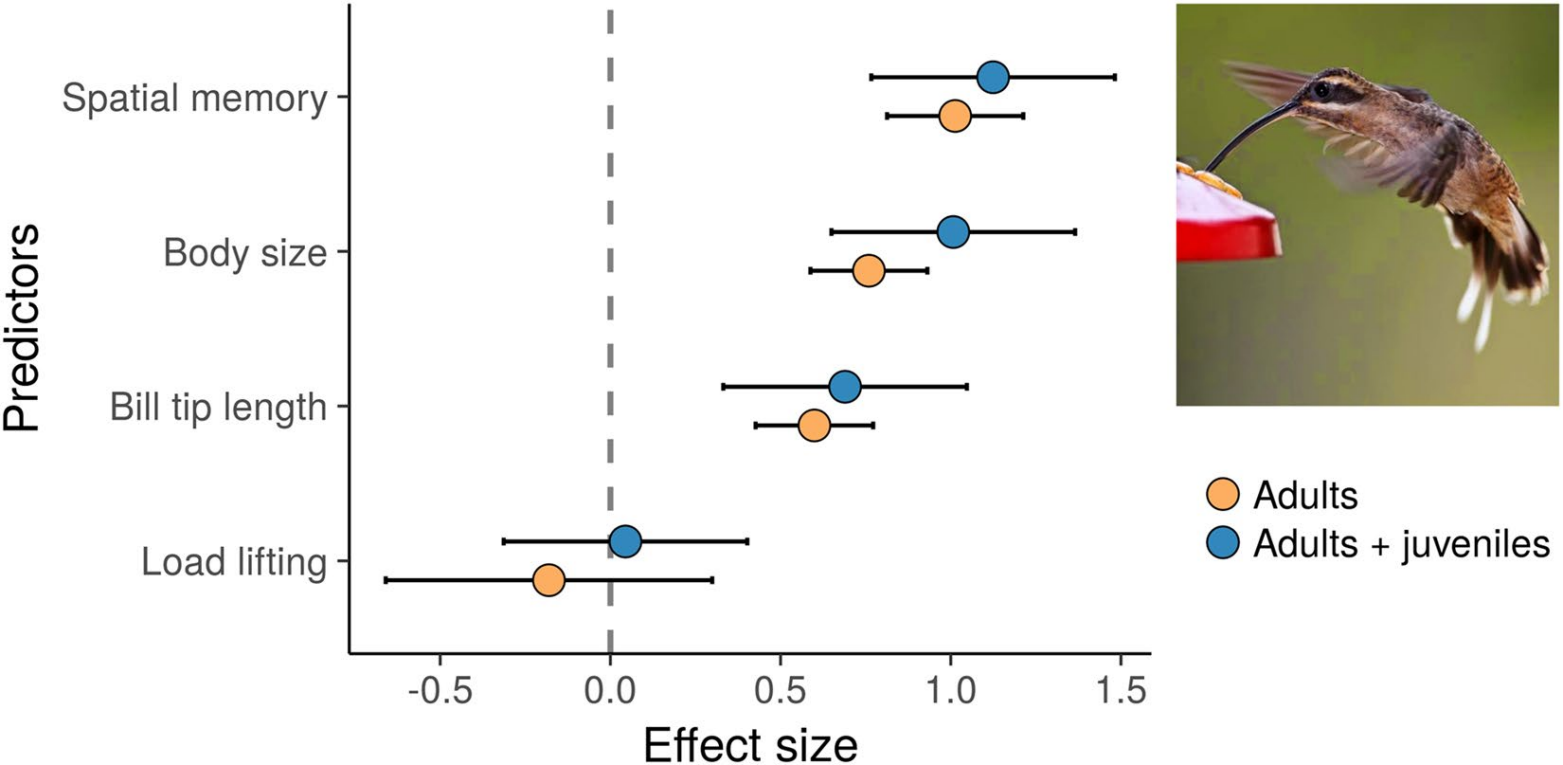
Standardized effect sizes and 95 confidence intervals of functional, morphological and cognitive traits for predicting territory acquisition in lekking long-billed hermit males. Effect sizes are shown for models on the complete data set, including juveniles (n = 30, blue markers) and the one on the subset of adult males (n = 20, orange markers). Effect sizes with confidence intervals that do not overlap with zero (highlighted by the vertical dashed line) were considered to have an effect on lek territory ownership. Photo (by David McDonald) shows a long-billed hermit visiting a feeder like those used in the spatial memory test.

**Figure 2:**
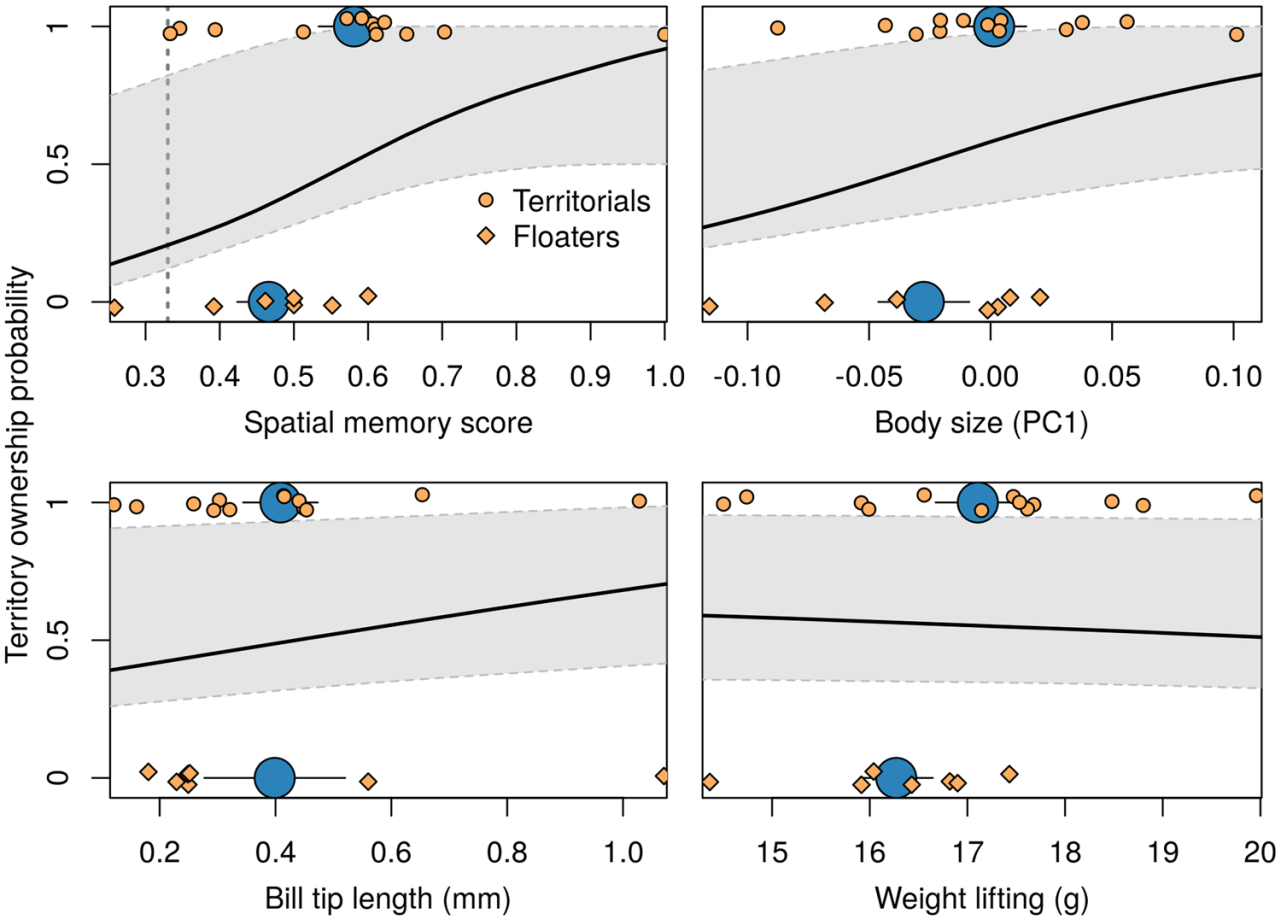
Predicted probability of territory ownership (black line) for four phenotypic traits from adult male long-billed hermits. Observed values for territorial and floater males are shown by orange circles and diamonds, respectively and the average (+/−S.E.) values are shown in blue. The 95% confidence intervals of the probabilities are shown in gray. Body size was calculated as the first principal component from a PCA on three morphological traits: body mass, total culmen, flattened wing length and central rectrice length. The vertical dotted line on the spatial memory graph shows the spatial memory performance expected by chance (e.g. if memory is not used).

Spatial memory, body size and bill tip length, but not load lifting, positively predicted the probability to acquire and defend a lek territory (Figs 1 and 2; Table 1; n = 30; mean number of trials per male = 38.2 +/− 26.2 SD). The first principal component used as a proxy for body size represented 54% of the variation in morphometric parameters. All morphometric parameters had positive loadings on the first component. The single best model, which accounted for 99% of the AICc weights, also included a positive effect of load lifting, although its effect size did not differ from zero (Tables 1 and S1). The model containing spatial memory, body size and bill tip length failed to converge and was excluded from the analysis. Qualitatively equivalent results were obtained after removing juvenile males: all predictors except for load lifting contribute to predict territory ownership (Table 1). In this case two models accounted for more than 95% of the AICc weights: one including all four predictors and another one including bill tip length, spatial memory and body size. Confidence intervals overlapped for all three predictors (Fig. 1, Table S1). However, differences between effect sizes among predictors became more apparent after removing juveniles. Bill tip length, but no other predictors, correlated significantly with age (Table S3). Spatial memory scores were not affected by total number of trials, mean daily number of trials nor mean time intervals between visits (Fig. S2).

**Table 1:** Effect sizes and 95% confidence intervals for parameters predicting lek territory ownership and song structure in long-billed hermits. Effect sizes that did not overlap with zero are shown in bold.

Response	Data set	Predictor	Effect size	Lower CI	Upper CI	Sample size
Territory ownership	Adults & juveniles	**Bill tip length**	**0**.**6895**	**0**.**3316**	**1**.**0473**	30
		**Body size (PC1)**	**1**.**0074**	**0**.**6496**	**1**.**3653**	30
		Load lifting	0.0443	−0.3135	0.4022	30
		**Spatial memory**	**1**.**1244**	**0**.**7665**	**1**.**4822**	30
	Adults	**Bill tip length**	**0**.**5993**	**0**.**4267**	**0**.**7720**	20
		**Body size (PC1)**	**0**.**7594**	**0**.**5876**	**0**.**9312**	20
		Load lifting	−0.1805	−0.6601	0.2991	20
		**Spatial memory**	**1**.**0130**	**0**.**8129**	**1**.**2131**	20
Song deviation	Adults & juveniles	Body size (PC1)	−0.2091	−0.0031	0.0043	26
		Signal-to-noise ratio	−0.0045	−0.3271	0.3425	26
	Adults	Signal-to-noise ratio	−0.0039	−0.0031	0.0043	25
		Spatial memory	−0.3971	−0.3271	0.3425	25
Song consistency	Adults & juveniles	**Signal-to-noise ratio**	**−0**.**0069**	**−0**.**0007**	**0**.**0037**	26
		Spatial memory	0.3144	−0.0364	0.3642	26
	Adults	**Signal-to-noise ratio**	**−0**.**0069**	**−0**.**0119**	**−0**.**0017**	26
		**Spatial memory**	**0**.**3144**	**0**.**1016**	**0**.**5248**	26
Mean frequency	Adults & juveniles	Body size (PC1)	0.0402	−0.1228	0.2011	137
		Signal-to-noise ratio	0.0004	−0.0014	0.0022	137
	Adults	Body size (PC1)	0.0383	−0.1361	0.2125	123
		Signal-to-noise ratio	0.0003	−0.0015	0.0021	123
Lowest frequency	Adults & juveniles	**Body size (PC1)**	**−0**.**3085**	**−0**.**6101**	**−0**.**0069**	137
		**Signal-to-noise ratio**	**−0**.**0043**	**−0**.**0076**	**−0**.**0010**	137
	Adults	Body size (PC1)	−0.2258	−0.5543	0.1026	123
		**Signal-to-noise ratio**	**−0**.**0041**	**−0**.**0075**	**−0**.**0007**	123
Duration	Adults & juveniles	Body size (PC1)	−0.1395	−0.3747	0.0930	137
		Signal-to-noise ratio	0.0008	−0.0017	0.0034	137
	Adults	Body size (PC1)	−0.1362	−0.3920	0.1224	123
		Signal-to-noise ratio	0.0013	−0.0014	0.0039	123

**Figure 3:**
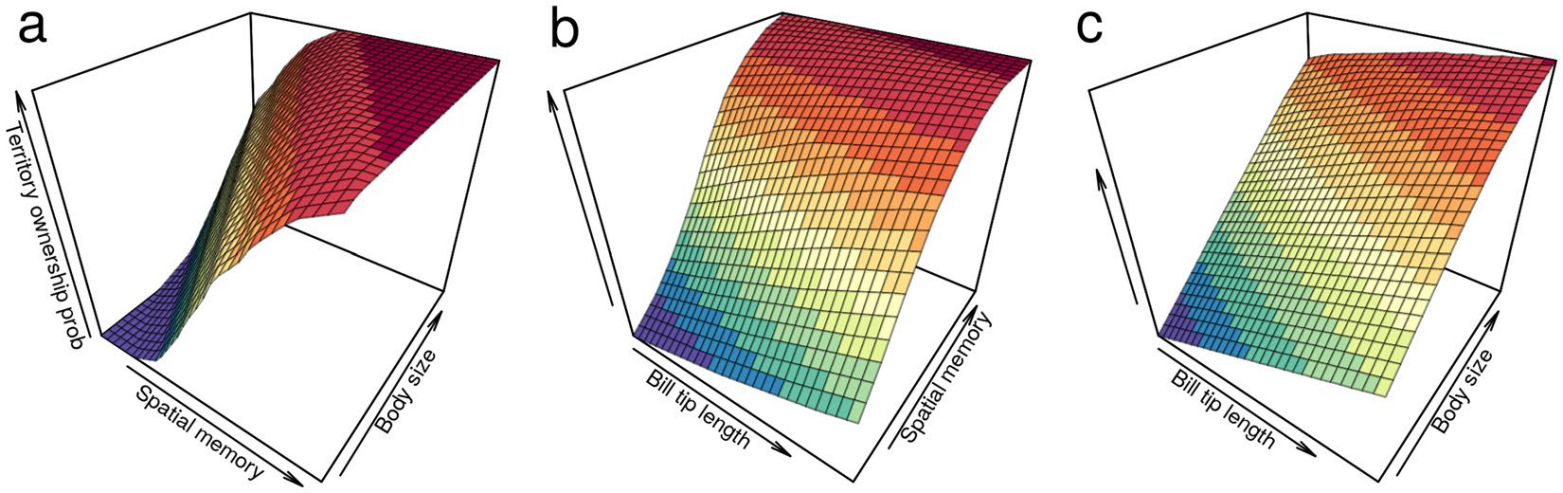
Three-dimensional surfaces of the predicted probability of territory ownership (z axis) for the combinations of the three traits that showed a significant effect. Probability of territory ownership was predicted based on the best mixed-effects model in the model selection procedure (two predictors at a time in the x and y axes). Values were predicted for all possible combinations of predictors (across 30 equally spaced values within the observed range) and random effect levels.

Spatial memory played a more important role for territory ownership (Fig. 3). The three-dimensional surface plot with spatial memory and body size as predictors showed a rapid increase in territory ownership probability with higher spatial memory, even at small body sizes (Fig. 3a). However, the model predicted only a moderate probability increase with larger body size at low spatial memory (Fig. 3a). Both spatial memory and body size showed a stronger effect on predicting territory ownership compared to bill tip length (Fig. 3a–c), although only spatial memory reached the highest probability at the smallest bill tip length (Fig. 3b), further supporting its higher relevance in territory ownership.

**Figure 4:**
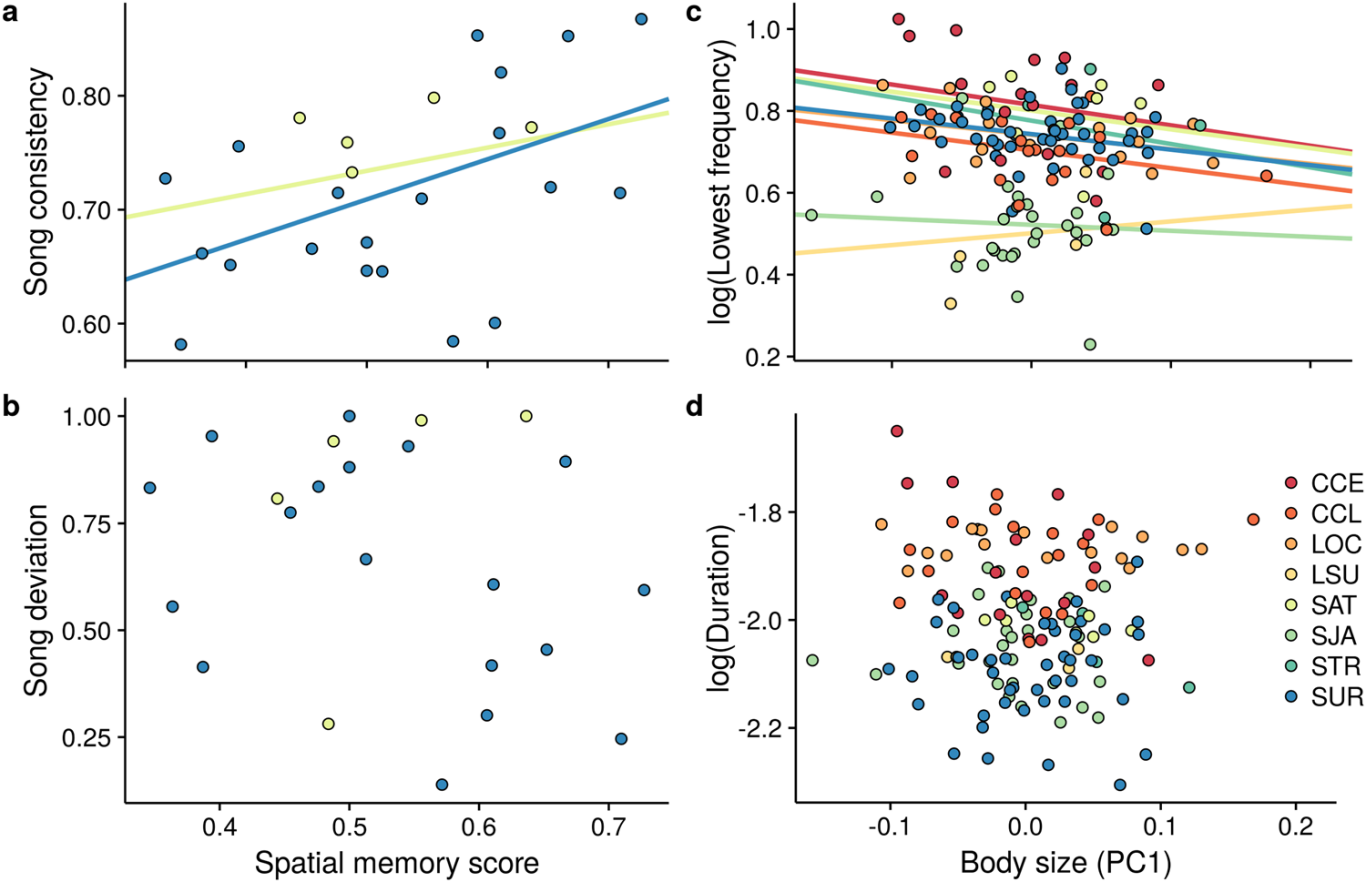
Relationships between song structure parameters and traits affecting lek territory ownership in long-billed hermits. Each individual is represented by a single data point and colors indicate the lek of origin. Spatial memory score was positively correlated with song consistency (**a**) but not with deviation (**b**) (only two leks are shown as only those had sufficient samples to support regression lines). Body size, correlated with song lowest frequency (**c**) but not with song duration (**d**).

**Figure 5:**
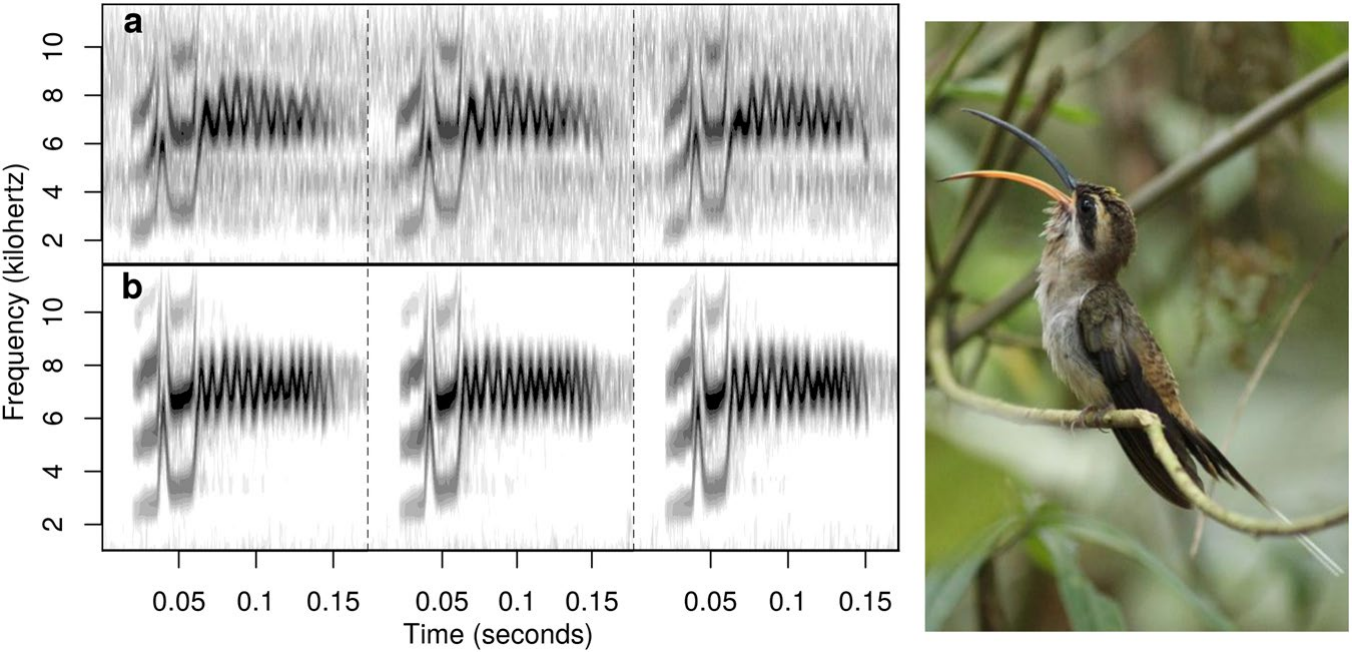
Spectrograms of long billed hermit vocalizations for males with (**a**) low (mean cross-correlation: 0.66) and (**b**) high song consistency (mean cross-correlation: 0.86) that share the same song type. The graph shows three of the five songs with the highest signal-to-noise ratio that were used in the song consistency analysis for the two individual. Photo (by Jeremy Gatten) illustrates a territorial long-billed hermit singing on a lek perch.

Spatial memory positively correlated with song consistency (i.e. within individual consistency) but not with song deviation (i.e. the deviation of an individual’s song from local song types) in adult territory owners (Fig. 4a,b, Table 1). The first principal component on morphological parameters used to characterize body size showed a significant negative correlation with lowest frequency (Figs 4d and 5; Table 1). Models predicting duration or mean frequency of songs did not performed better than the null (intercept-only) model (Fig. 4c and Table S1) and their effect sizes did not differ from zero (Table 1).

## Discussion

We studied the link between a cognitive ability, spatial memory, and a critical element for mating success in lekking species: the ability to acquire and defend a lek territory. We found that free living male long-billed hermits with higher cognitive performance were more likely to own lek territories and also were able to produce more consistent song, the primary signal of territory ownership. The results suggest a stronger effect of spatial memory compared to physical condition parameters traditionally associated to mating success and dominance^52,53^, particularly in lekking species^54–56^. Overall, our findings are consistent with a key role for cognition in mating in this species.

Spatial memory could be linked to territory ownership by three non-mutually exclusive mechanisms. The obvious one is that the higher foraging efficiency provided by spatial memory prowess allows males to spend less time foraging and more time on territorial defense. Evidence suggests that lek attendance involves important energetic costs^57,58^, creating an ‘endurance rivalry’^5,59^ in which spending more time at the lek improves territorial acquisition and reproductive output^55,56^. A second mechanism involves more efficient territorial defense facilitated by a better spatial memory. Lek territories in long-billed hermits are composed of several inconspicuous branches used as singing perches that are dispersed across ~150 m2^50^. Lekking males usually interact with other territorial males at these perches, which are visited even in the absence of owners^50^. Hence, lekking behavior seems to require significant spatial information processing, akin to the one demanded by foraging. Alternatively, better spatial memory could enhance foraging, and thus energy intake, improving body condition, which is also important for lek territoriality. However, spatial memory did not correlate with any of the condition parameters measured (discussed below).

An alternative explanation involves an improvement in spatial memory as a result of individuals adopting cognitively demanding territorial behavior. Nonetheless, this seems unlikely given that both territorials and floaters frequently visit the territory perches from other individuals^50^, which suggests that both types of males face similar cognitive challenges. Furthermore, spatial memory would be expected to improve with experience in territorial behavior. If so, the observed pattern would be a by-product of the experience gained by older individuals. Only bill tip length, however, showed a significant positive correlation with age (Table S3), an expected result previously documented in this species^46^. Spatial memory showed a non-significant negative correlation with age (Fig. S2), indicating no improvement throughout individual’s lifetime. In addition, the direction and relative magnitude effect of predictors remained unchanged after removing juveniles (although effect size differences among predictors became more apparent), further supporting a negligible role of age.

Unsuccessful foraging visits during the return phase might have little adaptive relevance if (1) they are not due to poorer spatial memory but rather to an alternative foraging strategy or (2) if the cost of missing rewarding flowers is small. Hummingbirds tend to adopt a “switch” strategy (i.e. trying a different resource next time) only when resources are depleted^60,61^. However, the feeders used in our experiment represented a non-depletable resource. Given the considerable habituation period, we would expect hummingbirds to adopt a “stay learning” foraging strategy, in which coming back to the rewarding feeder provides the highest payoff. Long-billed hermits visit many different flowering plants^50^ covering a wide range of nectar volumes, and daily patterns of nectar production^62^ suggesting that a plastic foraging strategy is also expected in this species. The cost of one error in one inflorescence is probably minimal, but the cumulative cost of mistaking rewarding flowers in several flowers during several foraging trips per day, in an 8 month breeding season, could be a significant disadvantage. Furthermore, the inflorescences visited by long-billed hermits at the study site can have several hundreds of flowers^62^, making a “trial-an-error” strategy inefficient.

Natural selection is predicted to optimize the combination of cognitive and non-cognitive phenotypic traits^63^. Furthermore, several combinations could provide similar fitness outcomes. Since advanced cognitive abilities involve energetic and developmental costs, they could generate trade-offs with other fitness related traits^2,3^. In this study individuals that performed better in the spatial memory task did not have larger body size or weapons, suggesting that different combinations of cognitive and non-cognitive traits can lead to the same outcome: territory ownership. If true, then advanced spatial memory, large body size and big weapons could all represent alternative (although not completely independent) strategies for intra-sexual competition (e.g. several combinations of body size and spatial memory that all generate the highest probability of territory ownership as shown in Fig. 3a). This finding also indicates that low cognitive performance does not affect self-maintenance. Hence, higher cognitive performance seems to be only favored by the competitive advantage in obtaining resources critical to reproduction, further supporting the role of sexual selection in promoting advanced spatial memory.

Research on cognition and sexual selection has mostly focused on testing their putative link and little is known on the relative importance of cognitive versus other types of traits. Our study provided a direct comparison between common phenotypic targets of sexual selection and a key cognitive trait: spatial memory. Body size is a common predictor of dominance and reproductive success in birds and other taxa^51,53^ a pattern also found in lekking species^54–56^. Weapon size is also related to male-male dominance and access to mates^64^ and has been previously found to predict territory ownership in long-billed hermits^46^. Interestingly, our results show a stronger effect of spatial memory in territory ownership when compared to body and weapon size. A similar pattern was also observed in guppies, in which female’s mating preference was predicted by male performance at a spatial memory task but not by body size or coloration^14^. Further research comparing the effect of cognitive traits to other known targets of selection should provide a clearer picture of the adaptive value of cognition.

The correlation of song structure with two traits influencing territory tenure, body size and spatial memory supports the use of songs as a reliable or honest index signals of male competitive abilities. Honesty in index signals is given by physical, physiological or developmental constraints on organs directly involved in signal production^22–24^. Indeed, the negative relation between body size and vocal signal frequency is a classical example of an honest index signal^24^, due to its strong theoretical support^65,66^: low frequencies require longer and/or bigger vocal organs. This relationship is common in anurans^67^, although in birds it has been found mostly across species (i.e. bigger species show lower frequency vocalizations; e.g. ref.^68^) but rarely at the intra-specific level^69–71^. To our knowledge, the observed negative correlation between body size and lowest frequency in long-billed hermit songs provides the only known intraspecific example in a non-oscine (i.e. non-songbird) vocal learning bird.

The reliability of song consistency as an indicator of cognitive abilities might be due to shared developmental constraints^9,30^ or from a general positive association between cognitive traits (i.e. general intelligence^9^). The developmental constraint hypothesis has received significant empirical support^25^ but evidence remains equivocal for “general intelligence” in non-human animals^13^. As in songbirds and parrots, hummingbird song development is also influenced by learning^48,49^. Vocal learning seems to be particularly relevant in long-billed hermits as they are able to modify their songs throughout life^48^. In addition, the early stages of their vocal learning process are characterized by low song consistency^48^, suggesting a direct link between vocal learning and the song feature associated with spatial memory. Although the current evidence is insufficient to identify the mechanism enforcing honesty in these signals, our findings do show that songs encode information about a key cognitive trait on a song aspect that is clearly related to vocal learning^48^. Future work should focus on understanding the mechanical base of this link as well as the extent to which females make use of the information encoded by songs.

On the other hand, song deviation, which attempts to quantify vocal learning accuracy, did not correlate with spatial memory. Several causes might explain this result, including different developmental constraints, lack of “general intelligence”, or little pressure to conform with local song variants (although the existence of song neighborhoods suggests otherwise^48^). Alternatively, new birds might copy the song of a single individual instead of the average song of a singing neighborhood, which implies that learning accuracy should be measured as the similarity to the tutor song (a difficult proposition in wild birds). However, in this case its value as an honest signal would be unclear as females (or male opponents) would require an *a priori* knowledge of tutor songs, which seems unlikely given the fact that several males often share the same song type and tutors may no longer be present when a given male is singing^48^.

Load lifting, which reflects the power available for flight during aggressive interactions (ref.^72^, Video S1), was used as a measure of current physical condition. Despite elaborate flying maneuvers and chases being common in long-billed hermit territorial defense^46,50^, load lifting showed no detectable effect on territory ownership. This could be due to a stronger selection for long distance flying abilities in traplining species, although traplining and territorial hummingbird species do not seem to differ in their aerodynamic power (ref.^73^, but note that load lifting was not directly compared among the two types of species in this study). Alternatively, excess flying power could only be favored by female choice as elaborate flying displays are also used during female visitation^50^.

Lek territory ownership was used as a proxy for intra-sexual selection. High mating skew among territory owners due to female choice is common but not ubiquitous in lekking systems^54,56^. Nonetheless, it is clear that lek territoriality implies a reproductive advantage^1,5,56,74^, particularly in the absence of alternative strategies^75^. In lekking hummingbirds, territory ownership gives priority or exclusive access to females^50^. In addition, floaters are constantly attempting (unsuccessfully most of the time) to sing and display from perches of established territorial males, and in a few cases are able to gain control over a territory^50^, further supporting lek territoriality as a key resource for reproduction.

In conclusion, our findings are consistent with the longstanding idea that advanced cognitive abilities are important for mating success. Males that performed better at a spatial memory task were also more likely to be territorial, and the effect of spatial memory was more pronounced than those of physical traits generally considered important for mating success. Finally, spatial memory is reliably indicated by the structure of mating vocal signals, with males with strong spatial memory singing more consistent songs. Future work on this species should focus on the mechanisms linking cognition, territorial behavior and signaling and examine whether the same traits important in intra-sexual selection are favored by female choice.

## Methods

The study was conducted at 8 leks at La Selva Biological station (10°23′N, 84°1′W), Costa Rica, in two breeding seasons between March 2013 and June 2015. Birds were captured at the leks using standard 6 and 12 m mist nets (19 mm mesh size), ringed with an individually-numbered metal band and marked with foam tags that had unique three-color combinations, attached to the back of the bird with nontoxic eyelash glue (LashGrip-Ardell^76^). Behavioral observations were conducted to determine territory ownership. Perches of singing males were mapped using a 20 × 20 m grid system as reference. Then, a map of lek territories based on an initial observation period was used to identify areas for further intensive netting and observations to identify all territorial males. Territorial males were defined as those able to sing at the lek and defend their singing perches from other males (as described in 50) during the observation period. Conversely, “floaters” were males observed at the lek that were unable to sing or defend their singing perches from other individuals. This is a reliable method for assessing lek territoriality that has previously provided biologically meaningful results in this system^46^. We have found that the lek hierarchy (floaters vs territorials) remains stable during the study period at the lek (7–15 days) as well as in the few cases when we visited the same lek in different months during the same breeding season. Furthermore, changes in territory ownership tend to occur at the start of the breeding season (Dec–Jan) which then remains stable throughout the season^50^ suggesting that these are consistent categories and hence can have a significant effect on reproductive output.

### Morphometric measurements and sexing

We measured total culmen, flattened wing, central rectrice (i.e. tail feather), and body mass, which were used to estimate body size (see below). We also measure bill tip length, a key morphological trait for lek territory ownership in this species^46^ males have elongated, pointy bill tips that are used as weapons to stab opponents during aggressive interactions; territorial males have longer bill tips than floaters (ref.^46^, Video S1). Sex was genetically determined using a multiplex primer set that amplifies introns of the chromohelicase-DNA-binding gene that differ in size on the avian Z and W chromosomes^77^. Behavioral and morphological information were used to sex birds in which DNA analysis produced ambiguous results (SI).

### Spatial memory test

In this study we replicated a design previously used to assess spatial memory in free-living hummingbirds^38–42^. Spatial memory was measured on free-living long-billed hermits at 3 leks. We placed 900 ml commercial hummingbird feeders (Perky Pet #209B) at 1–2 locations surrounding leks. Feeders were modified to have a single opening for accessing “nectar”. Three feeders arranged in a row (one next to the other; Video S1) were made available at a consistent location for each lek. A single feeder was filled with clear sugar water (the rewarding feeder; ~100 ml of water with 25% sucrose concentration) while the other two contained only water. The experiment trial consisted in two phases: a search phase in which visiting individuals identified the rewarding feeder and a return phase (the first visit after identifying the rewarding feeder) in which the ability to recall the position of the rewarding feeder was evaluated (Video S1). Spatial memory was tested when the position of the rewarding feeder was the same in both phases. Only the first visit after the search phase (i.e. the first return) was evaluated. Each feeder configuration lasted in average 52 min (+/−27 S.D.) and birds visited the feeders in average every 35 min (+/−9 S.D.). Performance during the return phase was coded as a binary variable (0 = fail, 1 = success) in which successful visits required visiting the rewarding feeder first. As long-billed hermits do not defend feeding territories, many individuals could be observed visiting the feeders in a short period of time, Therefore, several individuals were tested simultaneously (i.e. with the same feeder setup during the same days). The position of the rewarding feeder was changed after most visiting individuals have completed the return phase and the experiment was run until most visiting marked individuals had completed at least 10 trials. However, we also included individuals with at least 5 trials in the analyses given sample size limitations. The individual spatial memory score was calculated as the average of the performance scores (range: 0–1; 1 means perfect performance). There was considerable variation in time intervals between visits, mean number of daily trials and total number of trials. Hence, we also evaluated whether these factors influenced the spatial memory performance.

### Load lifting

We determined the maximum weight that could be lifted by a hummingbird during vertical flight as a measure of current body condition (ref.^72^, Video S1). Load lifting measures the excess of flying power that is available for complex flights^72^ as the maneuvers and scape flights typical of hummingbird competitive interactions (ref.^78^, Video S1), including the ones employed during territorial defense in long billed hermits^46^. To measure load lifting we used a rubber harness connected to a nylon string placed around the neck of hummingbirds, with color beads attached along the string (Video S1). The average of the two flights with the highest load lifted was calculated for each trial as it provided the highest repeatability (SI, Fig. S2) and was then used as the load lifting score in subsequent analyses.

### Song analysis

Songs were recorded for territorial males at the leks during the same field seasons in which morphological/cognitive data was collected. The song of this species is composed of a single element that is continuously repeated throughout the singing bout (i.e. single song repertoire). The five recorded songs with the highest signal-to-noise ratio were used for each individual. From 15 frequency parameters measured on songs, we selected mean frequency and lowest frequency as these parameters showed the highest repeatability within individuals (higher than 0.8; calculated using mixed-effects models on log-transformed parameters with random intercepts for individual^79^) and low collinearity (Pearson r = 0.18). Only crystallized songs from adult males were analyzed as sub-song from juveniles are structurally more variable^48^.

We assessed the accuracy of vocal learning by estimating “song deviation”: the distance of an individual’s songs to the average vocal structure of its song neighborhood (i.e. subgroup of individuals that share the same song type). Vocal structure was defined by the tridimensional acoustic space of song types from a Sammon’s mapping non-metric multidimensional scaling (using the R package “MASS”^80^) on spectrographic cross-correlation coefficients (after converted to distance). Song deviation was estimated as the distance between the average song structure of an individual and the centroid of the acoustic space for that particular song type (i.e. the average song structure of all other individuals in its song neighborhood). We also assessed the association between spatial memory and “song consistency”, defined as the average cross-correlation coefficient among the individual’s songs. All acoustic analyses were performed using the R packages warbleR^81^ and seewave^82^.

### Statistical analyses

We used model selection on generalized linear mixed-effects models on the R packages ‘MuMIn’^83^ and ‘lme4’^84^ to determine the factors influencing territory ownership. We used Akaike Information Criteria corrected for small sample sizes (AICc) as a measure of model relative support, due to the small sample size in relation to the number of parameters. A null model of no effect was also included in each selection routine. The best model was selected as the one with the lowest AICc value that accounted for at least 95% of the AICc weights in the candidate set. If more than one model was included, then effect sizes (and 95% confidence interval) for each fixed effect were calculated using model-averaging. Effect sizes that did not overlap with zero were considered to have an effect on the response variable. Models were fitted with maximum likelihood, using a binomial error structure and a logit link function to account for the binary response variable (territorial or floater). Four predictors were included in the models: spatial memory score, load lifting, bill tip length and body size. Body size was calculated as the first axis from a principal component analysis on (within lek mean-centered^85^) morphometric parameters (total culmen, flattened wing, central rectrice, and body mass). Males are more likely to be floaters during their first year at leks. Hence, models for territory ownership were evaluated on a data set including adults and juveniles and on a subset including only adults. We further explore the effect of age by estimating the correlation between age and predictors affecting territory ownership using Pearson product-moment correlation coefficient (on z-score transformed variables). This approach allowed us to assess whether the observed patterns arose as a by-product of age related changes in predictors.

We further explored the relative role of traits affecting lek territoriality by estimating the probability of territory ownership predicted by the best model in the model selection procedure. The probability was obtained by predicting the response variable in our model for all possible combinations of predictors and random effect levels (i.e. leks). The joint effect of traits was graphically assessed by generating three-dimensional surface plots of the matrix of average probabilities for each combination of trait values.

We also evaluated whether traits influencing territory ownership were reflected in the structure of acoustic signals. We assessed the relation between body size on song duration and frequency as these are the most common acoustic features affected by body size (refs^23,69^, but see ref.^70^) (formant dispersion (i.e. the distribution of amplitude across the resonance frequencies of the vocal tract) has also been associated to body size^86,87^ but was not evaluated as long-billed hermit songs show little harmonic content). We used mixed-effect models to assess the relation between log-transformed acoustic parameters (low frequency, mean frequency, and duration) and body size. A single model selection procedure similar to those described above was run for each acoustic parameter as the response variable. The first principal component as derived in the territory ownership analysis was used as a proxy for body size (as fixed effect). The signal-to-noise ratio of each song was also included as a covariate (fixed effect). As random effects we included random intercepts for individual and lek. Only songs from adult territory owners were included in all acoustic analyses.

Additional details on field and statistical methods are provided in the supplementary information. All activities described were reviewed and authorized by the Institutional Animal Care and Use Committee at the New Mexico State University (IACUC-2011-020) and were performed under the research permits 152-2009-SINAC and 063-2011-SINAC from Costa Rican Ministerio del Ambiente y Energia, in accordance with their guidelines and regulations. Prior to the study, all subjects were captured via mist nets and handled briefly for morphometric measurement and marking with small colored tags before being released at site of capture. During the study itself the birds behaved naturally without interference from the investigators.

### Data availability statement

All relevant data are provided as supporting information files.

## Electronic supplementary material

Supplementary video

Supporting information

Data sets

## References

[1] MacLean EL (2016). Unraveling the evolution of uniquely human cognition. Proc Natl Acad Sci.

[2] Shettleworth, S. *Cognition, evolution, and behavior* (Oxford University Press, 2010).

[3] Johnston T (1982). D- Selective costs and benefits in the evolution of learning. Adv Stud Behav.

[4] Thornton A, Isden J, Madden JR (2014). Toward wild psychometrics: Linking individual cognitive differences to fitness. Behav Ecol.

[5] Andersson, M. *Sexual selection* (Princeton University Press, 1994).

[6] Darwin, C. *The descent of man and selection in relation to sex* (J. Murray, 1871).

[7] Croston R, Branch CL, Kozlovsky DY, Dukas R, Pravosudov VV (2015). Heritability and the evolution of cognitive traits. Behav Ecol.

[8] Dukas R (2004). Evolutionary biology of animal cognition. Annu Rev Ecol Syst.

[9] Boogert NJ, Fawcett TW, Lefebvre L (2011). Mate choice for cognitive traits: A review of the evidence in nonhuman vertebrates. Behav Ecol.

[10] Kroodsma DE (1980). Winter Wren singing behavior: a pinnacle of song complexity. Condor.

[11] Lukianchuk KC, Doucet SM (2014). Cooperative courtship display in Long-tailed Manakins C*hiroxiphia linearis:* predictors of courtship success revealed through full characterization of display. J Ornithol.

[12] Borgia G (1995). Complex male display and female choice in the spotted bowerbird: specialized functions for different bower decorations. Anim Behav.

[13] Thornton A, Lukas D (2012). Individual variation in cognitive performance: developmental and evolutionary perspectives. Philos Trans R Soc B Biol Sci.

[14] Shohet AJ, Watt PJ (2009). Female guppies P*oecilia reticulata* prefer males that can learn fast. J Fish Biol.

[15] Keagy J, Savard JF, Borgia G (2009). Male satin bowerbird problem-solving ability predicts mating success. Anim Behav.

[16] Keagy J, Savard JF, Borgia G (2011). Cognitive ability and the evolution of multiple behavioral display traits. Behav Ecol.

[17] van Horik JO, Madden JR (2016). A problem with problem solving: Motivational traits, but not cognition, predict success on novel operant foraging tasks. Anim Behav.

[18] Spritzer MD, Meikle DB, Solomon NG (2005). Female choice based on male spatial ability and aggressiveness among meadow voles. Anim Behav.

[19] Prokosch MD, Coss RG, Scheib JE, Blozis SA (2009). Intelligence and mate choice: intelligent men are always appealing. Evol Hum Behav.

[20] Cole EF, Quinn JL (2012). Personality and problem-solving performance explain competitive ability in the wild. Proc Biol Sci.

[21] Lindenfors P, Nunn CL, Barton RA (2007). Primate brain architecture and selection in relation to sex. BMC Biol.

[22] Gil D, Gahr M (2002). The honesty of bird song: Multiple constraints for multiple traits. Trends Ecol Evol.

[23] Searcy, W. A. & Nowicki, S. *The evolution of communication reliability and deception in signaling systems* (2005).

[24] Maynard Smith, J. & Harper, D. *Animal Signals* (Oxford University Press, 2003).

[25] Boogert NJ, Giraldeau LA, Lefebvre L (2008). Song complexity correlates with learning ability in zebra finch males. Anim Behav.

[26] Templeton C, Laland K, Boogert N (2014). Does song complexity correlate with problem-solving performance in flocks of zebra finches?. Anim Behav.

[27] Farrell TM, Weaver K, An YS, MacDougall-Shackleton SA (2012). Song bout length is indicative of spatial learning in European starlings. Behav Ecol.

[28] Boogert NJ, Anderson RC, Peters S, Searcy WA, Nowicki S (2011). Song repertoire size in male song sparrows correlates with detour reaching, but not with other cognitive measures. Anim Behav.

[29] Sewall KB, Soha JA, Peters S, Nowicki S (2013). Potential trade-off between vocal ornamentation and spatial ability in a songbird. Biol Lett.

[30] Anderson RC (2017). Song learning and cognitive ability are not consistently related in a songbird. Anim Cogn.

[31] Healy SD, Hurly AT (2003). Cognitive Ecology: Foraging in Hummingbirds as a Model System. Adv Study Behav.

[32] Wells DJ (1993). Muscle performance in hovering hummingbirds. J Exp Biol.

[33] Suarez RK (1992). Hummingbird flight: sustaining the highest mass-specific metabolic rates among vertebrates. Cell Mol Life Sci.

[34] Chai P, Dudley R (1995). Limits to vertebrate locomotor energetics suggested by hummingbirds hovering in heliox. Nature.

[35] Suarez RK, Gass CL (2002). Hummingbird foraging and the relation between bioenergetics and behaviour. Comp Biochem Physiol Part A Mol Integr Physiol.

[36] Gass CL, Garrison JSE (1999). Energy regulation by traplining hummingbirds. Funct Ecol.

[37] Gill F (1988). Trapline foraging by hermit hummingbirds: competition for an undefended, renewable resource. Ecology.

[38] González-Gómez PL, Vasquez RA (2006). A field study of spatial memory in S*ephanoides sephaniodes*. Ethology.

[39] González-Gómez PL, Vásquez RA, Bozinovic F (2011). Flexibility of foraging behavior in hummingbirds: The role of energy constraints and cognitive abilities. Auk.

[40] González-Gómez PL, Bozinovic F, Vásquez RA (2011). Elements of episodic-like memory in free-living hummingbirds, energetic consequences. Anim Behav.

[41] González-Gómez PL (2014). Cognitive ecology in hummingbirds: the role of sexual dimorphism and its anatomical correlates on memory. Plos One.

[42] Hurly TA (1996). Spatial memory in rufous hummingbirds: memory for rewarded and non-rewarded sites. Anim Behav.

[43] Jelbert SA, Hurly TA, Marshall RES, Healy SD (2014). Wild, free-living hummingbirds can learn what happened, where and in which context. Anim Behav.

[44] Feinsinger P, Colwell RK (1978). Community organization among neotropical nectar-feeding birds. Am Zool.

[45] Krakauer DC, Rodriguez-Girones MA (1995). Searching and Learning in a Random Environment. J Theor Biol.

[46] Rico-Guevara. A, Araya-Salas M (2015). Bills as daggers? A test for sexually dimorphic weapons in a lekking hummingbird. Behav Ecol.

[47] Araya-Salas M, Wojczulanis-Jakubas K, Phillips EM, Mennill DJ, Wright TF (2017). To overlap or not to overlap: context-dependent coordinated singing in lekking long-billed hermits. Anim Behav.

[48] Araya-Salas M, Wright T (2013). Open-ended song learning in a hummingbird. Biol Lett.

[49] Baptista L, Schuchmann K (1990). Song learning in the Anna hummingbird (Calypte anna). Ethology..

[50] Stiles, F. G. & Wolf, L. L. Ecology and evolution of lek mating behavior in the long-tailed hermit hummingbird. O*rnithol Monogr* **27** (1979).

[51] Ellis L (1995). Dominance and reproductive success among nonhuman animals: a cross-species comparison. Ethol Sociobiol.

[52] Otter, K. A. *Ecology and Behavior of Chickadees and Titmice: An integrated approach* (2007).

[53] Hunt J, Breuker CJ, Sadowski JA, Moore AJ (2009). Male–male competition, female mate choice and their interaction: determining total sexual selection. J Evol Biol.

[54] Fiske P, Rintamäki PT, Karvonen E (1998). Mating success in lekking males: a meta-analysis. Behav Ecol.

[55] Wiley RH (1991). Lekking in birds and mammals: behavioral and evolutionary issues. Adv Study Behav.

[56] Höglund, J. & Alatalo, R. *Leks* (Princeton University Press, 2014).

[57] Höglund J (1992). The costs of secondary sexual characters in the lekking great snipe (G*allinago media)*. Behav Ecol Sociobiol.

[58] Cowles SA, Gibson RM (2014). Displaying to females may lower male foraging time and vigilance in a lekking bird. Auk.

[59] Clark CJ (2012). The role of power versus energy in courtship: what is the “energetic cost” of a courtship display?. Anim Behav.

[60] Cole S, Hainsworth FR, Kamil AC, Mercier T, Wolf LL (1982). Spatial learning as an adaptation in hummingbirds. Science.

[61] Hurly TA, Healy SD (1996). Memory for flowers in rufous hummingbirds: location or local visual cues?. Anim Behav.

[62] Stiles F (1975). Ecology, flowering phenology, and hummingbird pollination of some Costa Rican Heliconia species. Ecology.

[63] Rowe C, Healy SD (2014). Measuring variation in cognition. Behav Ecol.

[64] Emlen DJ (2008). The evolution of animal weapons. Annu Rev Ecol Evol Syst.

[65] Fletcher NH (2004). A simple frequency-scaling rule for animal communication. J Acoust Soc Am.

[66] Bradbury, J. & Vehrencamp, S. *Principles of animal communication* (Sinauer Associates Inc, 2011).

[67] Gerhardt, H. & Huber, F. *Acoustic communication in insects and anurans: common problems and diverse solutions* (University of Chicago Press, 2002).

[68] Medina-García A, Araya-Salas M, Wright T (2015). Does vocal learning accelerate acoustic diversification? Evolution of contact calls in Neotropical parrots. J Evol Biol.

[69] Hall ML, Kingma SA, Peters A, Blevins. W, Vanbroeckhoven C (2013). Male songbird indicates body size with low-pitched advertising songs. PLoS One.

[70] Cardoso G, Mamede A, Atwell J, Mota P (2008). Song frequency does not reflect differences in body size among males in two oscine species. Ethology.

[71] Fitch W (2000). Skull dimensions in relation to body size in nonhuman mammals: the causal bases for acoustic allometry. Zool Complex Syst.

[72] Altshuler DL, Dudley R, Mcguire JA (2004). Resolution of a paradox: Hummingbird flight at high elevation does not come without a cost. Proc Natl Acad Sci.

[73] Altshuler DL, Stiles FG, Dudley R (2004). Of hummingbirds and helicopters: hovering costs, competitive ability, and foraging strategies. Am Nat.

[74] Wong BBM, Candolin U (2005). How is female mate choice affected by male competition?. Biol Rev.

[75] Sinervo B, Zamudio KR (2001). The evolution of alternative reproductive strategies: fitness differential, heritability, and genetic correlation between the sexes. J Hered.

[76] Stiles FG, Wolf LL (1973). Techniques for color-marking hummingbirds. Condor.

[77] Han JI, Kim JH, Kim S, Park SR, Na KJ (2009). A simple and improved DNA test for avian sex determination. Auk.

[78] Clark CJ (2009). Courtship dives of Anna’s hummingbird offer insights into flight performance limits. Proc R Soc B.

[79] Nakagawa S, Schielzeth H (2010). Repeatability for Gaussian and non-Gaussian data: a practical guide for biologists. Biol Rev.

[80] Venables, W. N. & Ripley, B. D. *Modern applied statistics with S-PLUS* (Springer, 2002).

[81] Araya-Salas M, Smith-Vidaurre G (2017). warbleR: An R package to streamline analysis of animal acoustic signals. Methods Ecol Evol.

[82] Sueur J, Aubin T, Simonis C (2008). Equipment review: seewave, a free modular tool for sound analysis and synthesis. Bioacoustics.

[83] Barton, K. MuMIn: Multi-model inference. R package version 1.9.13 (2015).

[84] Bates D, Maechler M, Bolker B, Walker S (2016). Package “lme4”: Linear Mixed-Effects Models using “Eigen” and S4. R package version.

[85] van de Pol M, Wright J (2009). A simple method for distinguishing within-versus between-subject effects using mixed models. Anim Behav.

[86] Reber SA (2017). Formants provide honest acoustic cues to body size in American alligators. Sci Rep.

[87] Budka M, Osiejuk TS (2013). Formant frequencies are acoustic cues to caller discrimination and are a weak indicator of the body size of corncrake males. Ethology.

