# Supplementary material for: Spatial memory is as important as weapon and body size for territorial ownership in a lekking hummingbird
Source: Sci Rep. 2018 Jan 31;8:2001. doi: 10.1038/s41598-018-20441-x (PMC5792557; doi:10.1038/s41598-018-20441-x)
Supplement: Supplementary file 2 — Supporting information [file 41598_2018_20441_MOESM2_ESM.doc]

**Spatial memory is as important as weapon and body size for territorial ownership in a lekking hummingbird**

Marcelo Araya-Salas^1,2,6^ *, Paulina Gonzalez-Gomez^3^, Katarzyna Wojczulanis-Jakubas^4^, Virgilio López III^5^, Timothy F. Wright^6^

1. Laboratory of Ornithology, Cornell University, Ithaca, NY, U.S.A.

2. Escuela de Biología, Universidad de Costa Rica, San Pedro, San José, Costa Rica.

3. Department of Neurobiology, Physiology and Behavior, University of California Davis, CA, USA.

4. Department of Vertebrate Ecology and Zoology, University of Gdansk, Gdansk, Poland.

5 Department of Kinesiology, University of Connecticut, CT, USA.

6. Department of Biology, New Mexico State University, Las Cruces, NM, USA.

* Correspondence: M. Araya-Salas, Laboratory of Ornithology, Cornell University, 159 Sapsucker Woods Rd, Ithaca, NY 14850, U.S.A.

E-mail address: araya-salas@cornell.edu

**Supporting Information**

**Methods**

We measured total culmen (digital caliper, ±0.01 mm), flattened wing and central rectrice length (stopped wing ruler, ±0.5 mm), and body mass (digital scale ±0.01 g), which were used to estimate body size. Sex related morphometric variation has been reported for this species (1, 2). A function including body mass, total culmen and central rectriz length produced the best classification (100% of females, 94% of males; n males = 148, n females = 13). To measure bill tip length we took pictures of the lateral view of bill tips with a digital camera (Panasonic Lumix DMC-S3) coupled to a field dissecting microscope (30× magnification, Video S1). Bill tips were measured on pictures following the procedure described in (1). Bill tip shape and puncture capability are also related to territory acquisition in this species, but were not included in this analysis (1). Nonetheless, bill tip length is strongly and positively associated to these other features. Hence, we focused our analysis on bill tip length as it provides a simple and direct measure of weapon size. Songs were recorded on a Marantz PMD 660 and a Sennheiser ME62 microphone on a parabolic antenna (53 cm diameter).

Birds were aged based on the amount of corrugations in the maxillary ramphoteca (the keratinous covering of the upper bill). Corrugations cover most of the maxilla in juveniles, but less than 10% in adults (2, 3). Behavioral observations at each lek were conducted from 7:00 to 11:00 h and from 14:00 to 17:00 h (during the activity peak of the species (2)) through a 5-10 day period (depending on weather) to determine territory ownership.

Blood was collected from captured individuals by venipuncture in the tarsus and stored on Whatman FTA micro-elute cards (GE Healthcare Life Sciences) and stored at room temperature. DNA was extracted using manufacturers protocol and PCR performed with the P0-P2-P8 primer set (conditions available upon request). PCR products were visualized on a 2% agarose gel and sex scored by the presence of Z and W specific products. In some cases PCR-based sexing produced ambiguous results due to low DNA template concentrations. Some of these ambiguous individuals were observed holding lek territories and hence were sexed as males, given that females do not display this behavior (2). On the remaining unsexed birds we used cross-validation quadratic discriminant analysis on morphometric parameters for sexing, using the package ‘DiscriMiner’ (4) in the R statistical environment (5). In the final data set 24 birds were sexed based on DNA, 3 on behavior, and 3 on morphometrics.

Feeders used in the spatial memory test had identical visual cues with the same size and amount of water, a red bottom, and a yellow plastic flower on the opening (Video S1). Initially, a single feeder was placed (at ~1.5 m height) during a recruiting period of 4-7 days. Only the first return was included in the analysis. Observations were made from behind vegetation at least 6 m away from the feeder. Visits were also filmed using one or two digital cameras (Fujifilm HS30 EXR). Cameras were placed at ~3 m from feeders. Videos were used to double check field identifications and resolve observations of rapid behaviors.

Load lifting seems to provide a better estimate of physical condition than other common measures. The high individual repeatability of load lifting indicated that an intrinsic individual feature of flight performance was consistently measured by this test (6). In addition, other measures of physical condition emphasize the differences in body mass of individuals as a measure of energy reserves (e.g. residuals of regressions on log-transformed body mass (7, 8)). Hummingbirds use their energy intake very quickly (9) which makes the relevance of energy reserves dubious, particularly for a non-migrant species adapted to little environmental seasonality. Furthermore, weight is quite variable in this species; long-billed hermits can easily ingest a fifth of their body weight in sugar water during handling (MAS pers. obs). Therefore, is likely that body mass residuals reflect the daily nectar intake rather than energy reserves during a season.

Load lifting was measured using a rubber harness connected to a nylon string placed around the neck of hummingbirds, with color beads attached along the string (Video S1). Thirteen equally spaced beads were used. The total weight of the harness, string and beads was 15.0 g. Flight trials were conducted in the field, in a collapsible butterfly rearing cage (0.6 x 0.6 m wide, 0.9 m height). Trials were conducted before taking morphometric measurements to minimize any potential effect of manipulation-related stress. Cage walls were covered with black cloth such that the only light source was the transparent plastic top of the cage. This design ensured that birds tended to flight vertically, as they tried to escape through the only “opening” in the cage. Birds were released on the cage floor, where they flew while lifting the beaded string until reaching a maximum load (Video S1). Several trials were made for each bird during a ~1 min period (depending on bird’s motivation). To accurately determine the beads that were lifted, we filmed the string at the bottom of the cage (Video S1), and subsequently analyzed the videos frame-by frame. The total weight (i.e. load) lifted at each trial was calculated as the cumulative weight of the lifted beads plus the bird body mass at the time of the experiment.

Typically, for a given load lifting experiment a small number of trials consisted of vertical flights, while in other trials birds flew sideways or for a very short time, and lifted considerably less beads compared to their best performance trials. This variation could be particularly problematic in experiments with just a few trials, in which birds might not have been able to reach their full potential. Hence, we assessed the minimum number of trials needed to have a reliable load lifting measure by estimating its repeatability on birds tested in at least 2 experiments (in different days). We used linear mixed-effects models to estimate repeatability as the fraction of trait variation due to differences between individuals (6, 10). We ran models with load lifting as response variable, time of the day and days between experiments as predictors (both as single predictors and together in a model with two predictors) and individual as a random factor (random intercept), but no interaction terms. An intercept-only model with no effect was also included. The Akaike Information Criteria corrected for small sample sizes (AICc) was used as a measure of model relative support. The null model showed the lowest AICc, indicating no significant of time related variables. Therefore, load lifting repeatability was calculated with an intercept-only model and individual as random factor (10). To estimate the minimum number of trials that reliably represents load lifting, we calculated the repeatability for data subsets generated by excluding experiments that did not reach a cut-off number of trials (cut-off range: 4-10 trials). We calculated repeatability on a single load lifting value per experiment. This single value was estimated five ways: the average of all lifting trials in the experiment, the highest load lifting trial, and the average of the two, three and four highest load lifting trials. Therefore, five repeatability measures were obtained at each trial number cut-off. We found that load lifting repeatability increased substantially when evaluating trials with at least 8 flights, although with little variation between averaging approaches (Fig. S1). Scores were averaged across experiments for individuals with more than one experiment. Repeatability was estimated using the R package “rptR” (11).

Model selection was used to determine the combination of predictors (without interactions, including an intercept-only model) that better explained the variation in the response variable for all statistical models conducted. The Akaike Information Criteria corrected for small sample sizes (AICc) was used as a measure of model relative support. The best models were selected as the lowest AICc models that accounted for at least 95% of the AICc weights in the candidate set (12). Model-averaged effect sizes (when more several models accounted for the 95% AICc weights) with a 95% confidence interval were calculated for each fixed effect. Effect sizes were standardized to allow comparison across predictors (13). We also inspected the distribution of residuals and collinearity of predictors as well as the overdispersion of the global models (i.e. the one containing all predictors). Effect sizes that did not overlap with zero were considered to have a significant effect.

For models predicting territory ownership, predictors (except for PC1 on morphology) were z-score transformed to improve normality of model residuals. We anticipated a positive effect of bill tip length on lek territoriality (1), but no other association between model parameters was expected a priori. Hence, we generated all possible models that contained bill tip length as predictor. No interaction terms were considered in the models. We also included an intercept-only (null) model of no effect in the candidate model set (n = 8 models, Table 1 & Table S1). Lek of origin was included as a random effect (random intercept). The analysis was also run on a data set excluding juveniles. In this case we excluded all data from lek SAT as no floaters remained after filtering. Surface plots were produced using the R package ‘plot3D’ (14). Other plots were produced using ‘ggplot2’ (15).

We also evaluated the effect of sample size (i.e. total number of visits), mean time intervals between visits and number of trials per day on spatial memory scores using model selection on mixed-effect models. Predictors were z-score transformed to improve normality of residuals. The null model (i.e. an intercept only model) showed the lowest AICc, indicating no significant effect of the these additional parameters in the performance of the spatial memory test (Table S2). Accordingly, confidence interval of effect sizes for all the parameters overlapped with zero.

Model selection on mixed-effect models was also conducted to assess the relation between spatial memory score and both song deviation and consistency. Average signal-to-noise ratio was used as covariate (fixed effect) as it varies in recordings made under field conditions and can influence measures of acoustic structure. We also included lek of origin and individual as random factors (random intercepts, Table S1). All acoustic analyses were conducted with the R packages “tuneR” (16), “seewave” (17) and “warbleR” (18).

**References**

1. Rico-Guevara, A. & Araya-Salas, M. Bills as daggers? A test for sexually dimorphic weapons in a lekking hummingbird. *Behav Ecol* **26**, 21–29 (2015).

2. Stiles, F.G. & Wolf, L.L. Ecology and evolution of lek mating behavior in the long-tailed hermit hummingbird. *Ornithol Monogr* **27** (1979).

3. Ortiz-Crespo, F.I. A new method to separate immature and adult hummingbirds. *Auk* **89**, 851–857 (1972).

4. Sanchez, G. DiscriMiner: tools of the trade for discriminant analysis. *R Packag version 1.29* (2013).

5. R Core Team. R: A language and environment for statistical computing. Computing, Vienna, Austria. *R Found Stat Comput Vienna, Austria*. (2015).

6. Bell, A.M., Hankison. S.J. & Laskowski, K.L. The repeatability of behaviour: a meta-analysis. *Anim Behav* **77**, 771–783 (2009).

7. Peig, J. & Green, A.J. New perspectives for estimating body condition from mass/length data: the scaled mass index as an alternative method. *Oikos* **118**, 1883–1891 (2009).

8. Peig, J. & Green, A.J. The paradigm of body condition: a critical reappraisal of current methods based on mass and length. *Funct Ecol* **24**, 1323–1332 (2010).

9. Carleton, S.A., Bakken, B.H. & Martínez del Rio, C. Metabolic substrate use and the turnover of endogenous energy reserves in broad-tailed hummingbirds (*Selasphorus platycercus*). *J Exp Biol* **209**, 2622–2627 (2006).

10. Nakagawa, S. & Schielzeth, H. Repeatability for Gaussian and non-Gaussian data: a practical guide for biologists. *Biol Rev* **85**, 935–956 (2010).

11. Schielzeth, H., Stoffel, M, & Nakagawa, S. rptR: Repeatability Estimation for Gaussian and Non-Gaussian Data. R package version 0.9.1. (2017).

12. Burnham, K.P., Anderson, D.R. & Huyvaert, K.P. AIC model selection and multimodel inference in behavioral ecology: some background, observations, and comparisons. *Behav Ecol Sociobiol* **65**, 23–35 (2011).

13. Hox, J.J. *Multilevel analysis: techniques and applications* (2010).

14. Soetaert, K. plot3D: plotting multi-dimensional data. *R Packag version 10-1* (2014).

15. Wickham, H. *ggplot2 Elegant graphics for data analysis* (2009).

16. Ligges, U., Krey, S., Mersmann, O. & Schnackenberg, S. tuneR: analysis of music. *R Packag version 1.21* (2014).

17. Sueur, J., Aubin, T. & Simonis, C. Equipment review: seewave, a free modular tool for sound analysis and synthesis. *Bioacoustics* **18**, 213–226 (2008).

18. Araya-Salas, M. & Smith-Vidaurre, G. warbleR: An r package to streamline analysis of animal acoustic signals. *Methods Ecol Evol* **8**, 184–191 (2017).

Table S1. Parameters used by the model selection procedures for mixed-effect models predicting lek territory ownership, and song structure (song duration, lowest frequency, mean frequency, song deviation and song consistency). Delta AICc, AICc weights and cumulative AICc weights are computed among models with the same predictor and data set (group of models separated by thicker vertical lines).

| Data set | Response | Predictor(s) | Random factor(s) | Degrees of freedom | Log likelihood | AICc | Delta AICc | AICc weight | Cumulative AICc weight |
| --- | --- | --- | --- | --- | --- | --- | --- | --- | --- |
| Adults & juveniles | Lek territory ownership | Spatial memory, body size (PC1), load lifting, bill tip length | Lek | 7 | -4.78 | 28.6 | 0 | 0.998 | 0.998 |
| Adults & juveniles | Lek territory ownership | Spatial memory, bill tip length | Lek | 5 | -15.15 | 42.8 | 14.15 | 0.001 | 0.999 |
| Adults & juveniles | Lek territory ownership | Intercept only model | Lek | 2 | -19.71 | 43.9 | 15.23 | 0 | 0.999 |
| Adults & juveniles | Lek territory ownership | Spatial memory, load lifting, bill tip length | Lek | 6 | -14.22 | 44.1 | 15.44 | 0 | 1 |
| Adults & juveniles | Lek territory ownership | Bill tip length | Lek | 4 | -18.02 | 45.6 | 17 | 0 | 1 |
| Adults & juveniles | Lek territory ownership | Body size (PC1), bill tip length | Lek | 5 | -17.11 | 46.7 | 18.07 | 0 | 1 |
| Adults & juveniles | Lek territory ownership | Load lifting, bill tip length | Lek | 5 | -17.77 | 48 | 19.39 | 0 | 1 |
| Adults & juveniles | Lek territory ownership | Body size (PC1), load lifting, bill tip length | Lek | 6 | -17.07 | 49.8 | 21.14 | 0 | 1 |
| Adults | Lek territory ownership | Spatial memory, body size (PC1), bill tip length | Lek | 5 | -2.42 | 19.1 | 0 | 0.868 | 0.868 |
| Adults | Lek territory ownership | Spatial memory, body size (PC1), load lifting, bill tip length | Lek | 6 | -2.26 | 23 | 3.86 | 0.126 | 0.994 |
| Adults | Lek territory ownership | Intercept only model | Lek | 2 | -12.95 | 30.6 | 11.48 | 0.003 | 0.997 |
| Adults | Lek territory ownership | Spatial memory, bill tip length | Lek | 4 | -11.07 | 32.8 | 13.67 | 0.001 | 0.998 |
| Adults | Lek territory ownership | Spatial memory, load lifting, bill tip length | Lek | 5 | -9.27 | 32.8 | 13.7 | 0.001 | 0.998 |
| Adults | Lek territory ownership | Bill tip length | Lek | 3 | -12.95 | 33.4 | 14.26 | 0.001 | 0.999 |
| Adults | Lek territory ownership | Load lifting, bill tip length | Lek | 4 | -11.97 | 34.6 | 15.48 | 0 | 1 |
| Adults | Lek territory ownership | Body size (PC1), bill tip length | Lek | 4 | -12.03 | 34.7 | 15.6 | 0 | 1 |
| Adults | Lek territory ownership | Body size (PC1), load lifting, bill tip length | Lek | 5 | -11.72 | 37.7 | 18.61 | 0 | 1 |
| Adults | Duration | Intercept-only model | Lek, individual | 4 | 76.74 | -144.9 | 0 | 0.571 | 0.5715 |
| Adults | Duration | Signal-to-noise ratio | Lek, individual | 5 | 76.78 | -142.7 | 2.21 | 0.189 | 0.7607 |
| Adults | Duration | Body size (PC1) | Lek, individual | 5 | 76.74 | -142.6 | 2.29 | 0.181 | 0.9421 |
| Adults | Duration | Body size (PC1), signal-to-noise ratio | Lek, individual | 6 | 76.78 | -140.3 | 4.58 | 0.058 | 1 |
| Adults | Lowest frequency | Signal-to-noise ratio | Lek, individual | 5 | 66.58 | -122.3 | 0 | 0.456 | 0.456 |
| Adults | Lowest frequency | Body size (PC1), signal-to-noise ratio | Lek, individual | 6 | 67.03 | -120.8 | 1.47 | 0.218 | 0.6742 |
| Adults | Lowest frequency | Intercept-only model | Lek, individual | 4 | 64.63 | -120.7 | 1.59 | 0.205 | 0.8797 |
| Adults | Lowest frequency | Body size (PC1) | Lek, individual | 5 | 65.25 | -119.6 | 2.66 | 0.12 | 1 |
| Adults | Mean frequency | Body size (PC1) | Lek, individual | 5 | 114.83 | -218.8 | 0 | 0.448 | 0.4477 |
| Adults | Mean frequency | Body size (PC1), signal-to-noise ratio | Lek, individual | 6 | 115.69 | -218.1 | 0.65 | 0.323 | 0.771 |
| Adults | Mean frequency | Signal-to-noise ratio | Lek, individual | 5 | 113.58 | -216.3 | 2.51 | 0.128 | 0.8989 |
| Adults | Mean frequency | Intercept-only model | Lek, individual | 4 | 112.2 | -215.8 | 2.98 | 0.101 | 1 |
| Adults | Song consistency | Spatial memory, signal-to-noise ratio | Lek, individual | 6 | 35.05 | -53.7 | 0 | 0.627 | 0.627 |
| Adults | Song consistency | Spatial memory | Lek, individual | 5 | 32.22 | -51.4 | 2.25 | 0.203 | 0.831 |
| Adults | Song consistency | Signal-to-noise ratio | Lek, individual | 5 | 31.54 | -50.1 | 3.6 | 0.104 | 0.934 |
| Adults | Song consistency | Intercept-only model | Lek, individual | 4 | 29.54 | -49.2 | 4.51 | 0.066 | 1 |
| Adults | Song deviation | Intercept-only model | Lek, individual | 4 | 0.24 | 9.5 | 0 | 0.597 | 0.597 |
| Adults | Song deviation | Spatial memory | Lek, individual | 5 | 0.83 | 11.5 | 1.98 | 0.222 | 0.818 |
| Adults | Song deviation | Signal-to-noise ratio | Lek, individual | 5 | 0.33 | 12.5 | 2.98 | 0.135 | 0.953 |
| Adults | Song deviation | Spatial memory, signal-to-noise ratio | Lek, individual | 6 | 1.03 | 14.6 | 5.09 | 0.047 | 1 |


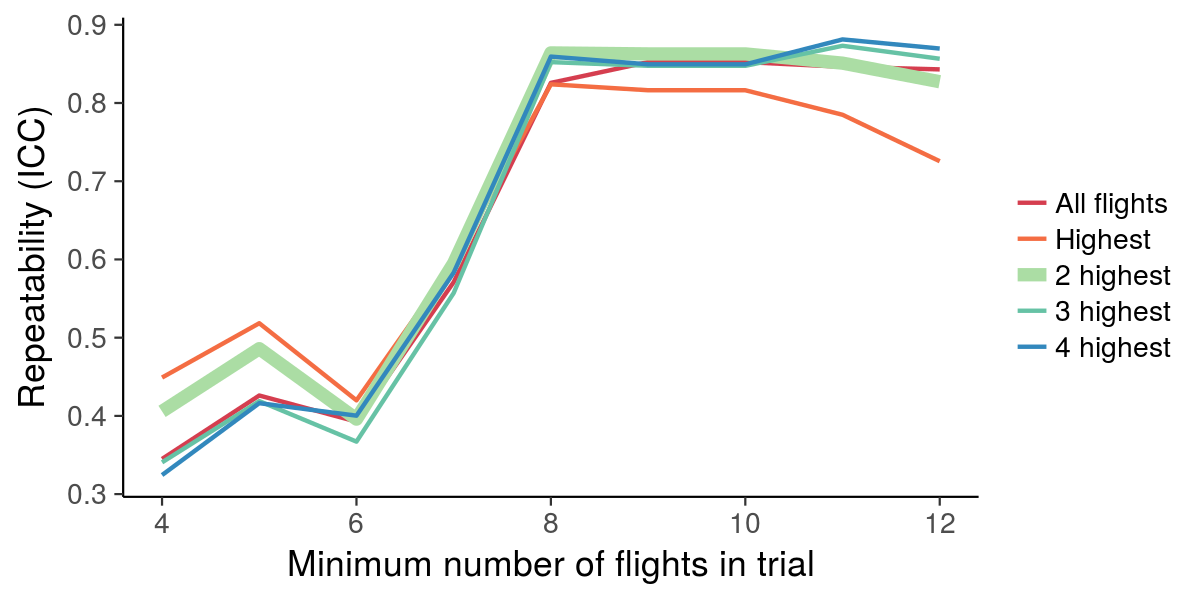


Fig. S1. Repeatability of load lifting performance by minimum number of trials per experiment for individuals measured in two or more experiments (in different days). Repeatability was calculated with the Intraclass Correlation Coefficient. For each number of trials cut-off, experiments that not reach the cut-off number of trials were excluded. A single load lifting score was calculated on five ways: the average of all lifting trials in the experiment, the highest load lifting trial, and the average of the two, three and four highest lifting trials (depicted by the line colors). Therefore, five repeatability measures were obtained at each trial number cut-off. Load lifting repeatability increased substantially after 8 flights. The average of the two flights with the highest load lifted was used as the load lifting performance measure (thicker yellow line).

Table S2. Parameters used by the model selection procedures for mixed-effect models assessing the influence of variation in feeder visitation in spatial memory score. Delta AICc, AICc weights and cumulative AICc weights are computed among models with the same predictor and data set.

| Response | Predictor(s) | Random factor | Degrees of freedom | Log likelihood | AICc | Delta AICc | AICc weight | Cumulative AICc weight |
| --- | --- | --- | --- | --- | --- | --- | --- | --- |
| Spatial memory | Intercept-only model | Individual | 8.18 | -9.9 | 0.000 | 0.385 | 8.18 | 0.385 |
|  | Inter-feeding interval |  | 8.51 | -8.2 | 1.678 | 0.166 | 8.51 | 0.551 |
|  | Trials per day |  | 8.32 | -7.8 | 2.056 | 0.138 | 8.32 | 0.688 |
|  | Total number of visits |  | 8.25 | -7.6 | 2.209 | 0.127 | 8.25 | 0.816 |
|  | Visits per day, total number of visits |  | 8.68 | -6.1 | 3.792 | 0.058 | 8.68 | 0.873 |
|  | Inter-feeding interval, total number of visits |  | 8.65 | -6.0 | 3.848 | 0.056 | 8.65 | 0.929 |
|  | Trials per day, inter-feeding interval, total number of visits |  | 8.55 | -5.8 | 4.049 | 0.051 | 8.55 | 0.980 |

Table S3. Pearson correlation coefficients and 95% confidence intervals (in parentheses) between parameters predicting lek territory ownership and age (after z-score transformation). Upper triangle of the matrix shows coefficients for the data set including both adults and juveniles, while lower triangle shows data for adults only. Significant correlations are shown in bold.

|  | Age | Body size (PC1) | Spatial memory | Bill tip length | Load lifting |
| --- | --- | --- | --- | --- | --- |
| Age | 1 | 0.02 (-0.35_0.37) | -0.11 (-0.45_0.26) | **0.60 (0.3_0.79)** | -0.30 (-0.60_0.06) |
| Body size (PC1) | 0.16 (-0.30_0.56) | 1 | -0.22 (-0.54_0.15) | -0.1 (-0.44_0.27) | **0.38 (0.03_0.65)** |
| Spatial memory | -0.33 (-0.67_0.13) | -0.29 (-0.65_0.17) | 1 | -0.26 (-0.56_0.12) | 0.09 (-0.28_0.44) |
| Bill tip length | **0.67 (0.32_0.86)** | -0.03 (-0.47_0.42) | -0.33 (-0.67_0.13) | 1 | -0.02 (-0.38_0.34) |
| Load lifting | 0.22 (-0.25_0.6) | **0.45 (0.01_0.74)** | 0.01 (-0.43_0.45) | 0.41 (-0.04_0.72) | 1 |


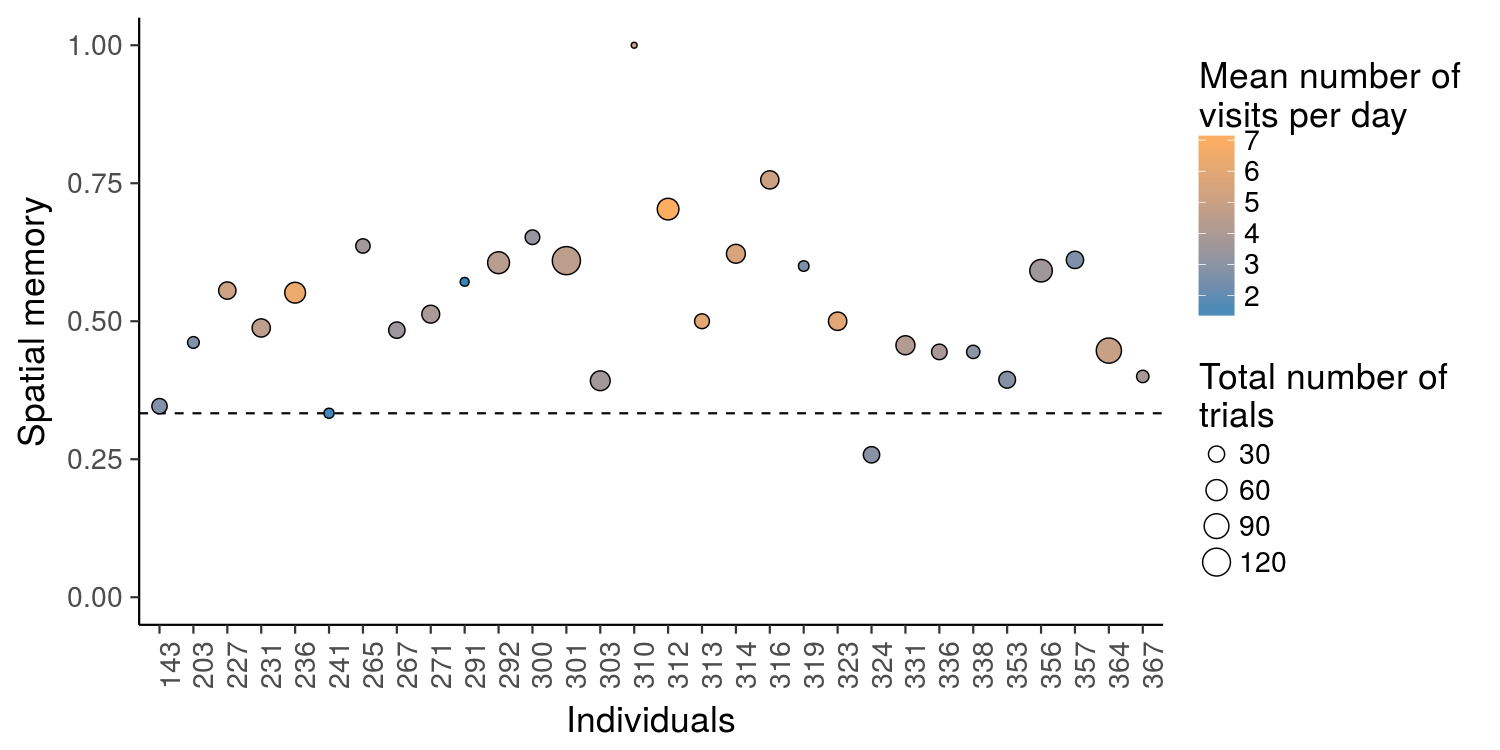
Fig. 2. Spatial memory score by individual The color scale shows the mean number of visits per day and the size scale the total number of trials. The dotted line shows the spatial memory score expected by chance for a three feeder experimental setting (i.e. one out of every three trials).
